# Association between *Toxoplasma gondii* infection and thyroid dysfunction: a case-control seroprevalence study

**DOI:** 10.1186/s12879-019-4450-0

**Published:** 2019-09-18

**Authors:** Cosme Alvarado-Esquivel, Agar Ramos-Nevarez, Carlos Alberto Guido-Arreola, Sandra Margarita Cerrillo-Soto, Alma Rosa Pérez-Álamos, Sergio Estrada-Martínez, Verónica Dayali Gutierrez-Martinez, Antonio Sifuentes-Alvarez, Eda Guadalupe Ramírez-Valles, Edith Contreras-Cisneros

**Affiliations:** 1 Biomedical Research Laboratory, Faculty of Medicine and Nutrition, Juárez University of Durango State, Avenida Universidad S/N, 34000 Durango, Mexico; 2 Clínica de Medicina Familiar, Instituto de Seguridad y Servicios Sociales de los Trabajadores del Estado, Predio Canoas S/N, 34079 Durango, Mexico; 3 Institute for Scientific Research “Dr. Roberto Rivera-Damm”, Juárez University of Durango State, Avenida Universidad S/N, 34000 Durango, Mexico; 4 Faculty of Chemical Sciences, Juárez University of Durango State, Avenida Universidad S/N, 34000 Durango, Dgo Mexico

**Keywords:** *Toxoplasma gondii*, Infection, Seroprevalence, Hypothyroidism, Hyperthyroidism, Epidemiology, Case-control study, Mexico

## Abstract

**Background:**

The association between *Toxoplasma gondii* infection and thyroid disease has been poorly studied. Therefore, we sought to determine the association between *T. gondii* seropositivity and thyroid dysfunction.

**Methods:**

We performed an age- and gender-matched case-control study of 176 patients suffering from hypothyroidism (*n* = 161) or hyperthyroidism (*n* = 15) and 528 control subjects without these diseases in a public hospital in Durango City, Mexico. Anti-*Toxoplasma* IgG antibodies were determined in sera from cases and controls using a commercially available enzyme-linked immunoassay.

**Results:**

Anti-*T. gondii* IgG antibodies were found in 11 (6.3%) of 176 patients suffering from thyroid dysfunction and in 48 (9.1%) of 528 control subjects (OR = 0.66; 95% CI: 0.33–1.31; *P* = 0.23). Stratification by two groups of age (50 years and younger, and 51 year and older) showed that the youngest group of patients with thyroid dysfunction had a significantly lower seroprevalence of *T. gondii* infection than its age- and gender-matched control group (1/83: 1.2% vs 23/257: 8.6%; OR = 0.12; 95% CI: 0.01–0.93; *P* = 0.01). This stratification also showed that the youngest group of patients with hypothyroidism had a significantly lower seroprevalence of *T. gondii* infection than its age- and gender matched control group (0/75: 0% vs 21/233: 9.0%; *P* = 0.003).

**Conclusions:**

Our results suggest that thyroid dysfunction is not associated with seropositivity to *T. gondii* in general; however, in young (50 years or less) patients, a negative association between infection and thyroid dysfunction and hypothyroidism was found. Further research to confirm this negative association is needed.

## Background

*Toxoplasma gondii* (*T. gondii*) is an obligated intracellular protozoan parasite [1]. *T. gondii* is responsible of morbidity and mortality worldwide [2–4]. This parasite causes a disease so called toxoplasmosis which often is not recognized and is inadequately managed [3]. Transmission of *T. gondii* usually occurs by the oral route, and raw or undercooked meat is an important transmission vehicle for *T. gondii* [5]. Humans can also acquire the infection by ingestion of environmental sporulated oocysts in contaminated food or water [6]. *T. gondii* can also be transmitted by organ transplantation [7], and blood transfusion [8]. In addition, *T. gondii* may cross the placenta of an infected pregnant women and probably infect the fetus congenitally [9]. Toxoplasmosis has a wide spectrum of clinical outcomes varying from asymptomatic to life-threatening disease [10, 11]. *T. gondii* may cause retinochoroiditis [11]. Reactivation of a latent infection in immune deficiency conditions can cause fatal toxoplasmic encephalitis [12]. Infection with *T. gondii* has been associated with psychiatric disorders, for instance: schizophrenia [13, 14], and mixed anxiety and depressive disorder [15].

Very little is known about infection with *T. gondii* in thyroid gland. In a study of nine autopsy cases of disseminated toxoplasmosis, researchers found involvement of the thyroid gland [16]. Anti-*T. gondii* antibodies have been associated with autoimmune thyroid diseases [17, 18]. Prior infection with *T. gondii* was associated with an elevation of autoantibodies to thyroid peroxidase [19]. Latent toxoplasmosis was associated with a mild increase of thyroid hormone production in pregnancy [20]. In addition, an impaired thyroid function was reported in murine toxoplasmosis [21]. Dubey et al., reported acute fatal systemic toxoplasmosis involving thyroid gland and other organs of a 13-month-old llama (*Llama glama*) [22]. Thus, we hypothesize that subjects with *T. gondii* infection may have thyroid gland involvement leading to thyroid dysfunction. In this study, we sought to determine the association between *T. gondii* seropositivity and thyroid dysfunction in people in Durango City, Mexico.

## Methods

### Study design and subjects studied

We performed an age- and gender-matched case-control study of 176 patients suffering from thyroid dysfunction and 528 subjects without thyroid dysfunction. Patients and controls were enrolled consecutively in a health campus of a public institution (Institute of Security and Social Services for the State Workers) in Durango City, Mexico. This health campus comprises a hospital (where the cases were obtained) and a clinic of family medicine (where the controls were obtained). Blood sampling was performed in the clinical laboratory at the clinic of family medicine from September 2015 to October 2018. Inclusion criteria for enrollment of cases in the study were: 1) patients suffering from hypothyroidism or hyperthyroidism attended in the Hospital “Dr. Santiago Ramón y Cajal” of the Institute of Security and Social Services for the State Workers in Durango City; 2) aged 18 years and older; and 3) who accepted to participate in the survey. Diagnosis of hypothyroidism was made based on the detection of an abnormally high thyroid-stimulating hormone and a low level of free thyroxin. Whereas diagnosis of hyperthyroidism was made based on the detection of an abnormally low thyroid-stimulating hormone and an elevated free thyroxin. We were unable to diagnose autoimmune thyroid disfunction because of a lack of laboratory tests to support this diagnosis. Disease in patients was not further classified as primary and secondary dysfunction. All patients had had symptoms of thyroid disfunction. The length of evolution was not determined. Of the 176 cases, 161 had hypothyroidism and 15 had hyperthyroidism. Of whom, 152 (86.4%) were females, and 24 (13.6%) were males. Cases were 18–81 years old (mean age: 50.40 ± 12.8). Controls were selected from people attending the clinical laboratory in the clinic of family medicine. Blood sampling in controls was not only used for this study but also for laboratory tests asked by family medicine physicians for medical checkups, diagnosis or follow ups of diseases. None of the controls reported an autoimmune disease. Of the 528 controls, 456 (86.4%) were females and 72 (13.6%) were males. Gender in controls was similar to that in cases (*P* = 1.00). Controls were 18–84 years old (mean age: 50.39 ± 12.8), and their ages were similar to those in cases (*P* = 0.98). No difference in residence (Durango State) among cases and controls was found (*P* = 0.43). Other general sociodemographic characteristics as area of residence (urban, suburban and rural), education, and socioeconomic status were not used for matching; however, enrollment of cases and controls at the same health institution was an additional characteristic for matching.

### Detection of anti-*T. gondii* IgG antibodies

A serum sample from each case and control was obtained and kept frozen at − 20 °C until analyzed. Detection of anti-*T. gondii* IgG antibodies was performed using a commercially available enzyme immunoassay kit: “*Toxoplasma* IgG” (Diagnostic Automation/Cortez Diagnostics Inc., Woodland Hills, CA, USA). This test provides qualitative and quantitative results and has a grey zone (equivocal). Results that fell in the grey zone were considered as negative. Negative and positive controls provided by the manufacturer were included in each run, and the test procedure was performed according to the manufacturer’s instructions. Researchers who performed the laboratory tests were blind to the clinical data of participants.

### Statistical analysis

Data was analyzed with the aid of the software SPSS version 15.0 and Epi Info 7. For calculation of the sample size, we used the following parameters: a reference seroprevalence of 6.1% [23] as the expected frequency of exposure in controls, a two-sided confidence level of 95%, a power of 80%, a 1:3 proportion of cases and controls, and an odds ratio of 2.5. The result of the sample size calculation was 157 cases and 469 controls. To assess age matching, we used the student’s *t*-test. To compare differences in seropositive rates among groups the chi square test and the Fisher exact test were used. Odds ratio (OR) and 95% confidence interval (CI) were calculated to assess the association between *T. gondii* infection and thyroid dysfunction. Statistical significance was set at a *P* value < 0.05.

## Results

Anti-*T. gondii* IgG antibodies were found in 11 (6.3%) of 176 patients suffering from thyroid dysfunction and in 48 (9.1%) of 528 control subjects. One of the negative results of cases fell in the gray zone of the enzyme immunoassay. No statistically significant difference (OR = 0.66; 95% CI: 0.33–1.31; *P* = 0.23) in anti-*T. gondii* IgG seroprevalence between cases and controls was found.

Stratification by sex did not show a difference in seroprevalence of *T. gondii* infection between cases and controls (Table 1). Whereas stratification by age groups showed that cases aged 31–50 years old had a significantly lower (1.4%) seroprevalence of *T. gondii* infection than control subjects (9.5%) of the same age group (OR = 0.13; 95% CI: 0.01–1.02; *P* = 0.02) (Table 1). Further stratification by sex and age groups in hypothyroidism patients showed that seroprevalence of *T. gondii* infection was significantly (*P* = 0.005) lower in patients with hypothyroidism aged 31–50 years than control subjects of the same age group (Table 2). Stratification by only two groups of age (50 years and younger, and 51 and older) showed that the youngest group of patients with thyroid dysfunction had a significantly lower seroprevalence of *T. gondii* infection than its age- and gender-matched control group (1/83: 1.2% vs 23/257: 8.6%; OR = 0.12; 95% CI: 0.01–0.93; *P* = 0.01). This stratification also showed that the youngest group of patients with hypothyroidism had a significantly lower seroprevalence of *T. gondii* infection than its age- and gender-matched control group (0/75: 0% vs 21/233: 9.0%; *P* = 0.003). No OR and 95% CI could be calculated in this comparison because a zero value was present in one cell of the 2 × 2 table. Seroprevalence of *T. gondii* did not increase with age in cases, controls, or both groups together (*P* = 0.40, *P* = 0.90, and *P* = 0.88, respectively). Mean age of *T. gondii* seropositive cases (59.73 ± 9.14) was significantly (*P* = 0.005) higher than the mean age of seropositive controls (49.52 ± 10.65).

**Table 1:** Stratification by sex and age in cases and controls and seropositivity rates to *T. gondii*

	Cases			Controls					
Characteristics	No. tested	Seropositivity to *T. gondii*		No. tested	Seropositivity to *T. gondii*		Odds ratio	95% confidence interval	*P* value
		No.	%		No.	%			
Sex									
Male	24	1	4.2	72	10	13.9	0.26	0.03–2.22	0.28
Female	152	10	6.6	456	38	8.3	0.77	0.37–1.59	0.48
Age (years old)									
≤ 30	12	0	0.0	37	2	5.4	1.00	–	1.00
31–50	71	1	1.4	220	21	9.5	0.13	0.01–1.02	0.02
> 50	93	10	10.8	271	25	9.2	1.18	0.54–2.57	0.66

**Table 2:** Stratification by sex and age in cases of hypothyroidism and controls and seropositivity rates to *T. gondii*

	Cases			Controls					
Characteristics	No. tested	Seropositivity to *T. gondii*		No. tested	Seropositivity to *T. gondii*		Odds ratio	95% confidence interval	*P* value
		No.	%		No.	%			
Sex									
Male	22	1	4.5	66	9	13.6	0.30	0.03–2.52	0.44
Female	139	9	6.5	417	34	8.2	0.77	0.36–1.66	0.52
Age (years old)									
≤ 30	10	0	0.0	31	2	6.5	–	–	1.00
31–50	65	0	0.0	202	19	9.4	–	–	0.005
> 50	86	10	11.6	250	22	8.8	1.36	0.61–3.0	0.44

Concerning clinical diagnosis, 10 (6.2%) of 161 patients with hypothyroidism and 43 (8.9%) of 483 age- and gender-matched control subjects were positive for anti-*T. gondii* antibodies (*P* = 0.28). Whereas one (6.7%) of 15 patients with hyperthyroidism and 5 (11.1%) of 45 age- and gender-matched control subjects were positive for anti-*T. gondii* antibodies (*P* = 1.00). Patients suffering from hypothyroidism had a similar seroprevalence of *T. gondii* infection to patients suffering from hyperthyroidism (*P* = 1.00).

The frequency of high (> 150 IU/ml) anti-*T. gondii* IgG antibody levels in cases was similar to the one found in controls (2/176 vs 19/528; *P* = 0.12). In addition, the frequency of high (> 150 IU/ml) anti-*T. gondii* IgG antibody levels in hypothyroidism patients was similar to the one found in their controls (2/161 vs 19/483; *P* = 0.12).

## Discussion

Whether infection with *T. gondii* is associated with thyroid dysfunction is largely unknown. Infection with *T. gondii* in the thyroid gland has been demonstrated in humans [16] and a llama [22]. However, information about the impact of this parasite on thyroid function is scanty. Therefore, by using an age- and gender-matched case-control seroprevalence study design we sought to determine the association between seropositivity to *T. gondii* and thyroid dysfunction in people attending a public hospital in the northern Mexican city of Durango. In the present study we did not only match cases and controls by age and gender but also by health institution. Both cases and controls were enrolled in the same public institution. This fact makes the population groups more uniform. We found that patients with thyroid dysfunction had a comparable seroprevalence of *T. gondii* infection to control subjects in general (6.3 and 9.1%, respectively). A previous study reported a 6.1% seroprevalence of *T. gondii* infection in general population in Durango City [23]. However, these seroprevalences should be compared with care since mean age (37.04 ± 16.14) in subjects in that study was lower than the mean age (50.40 ± 12.8) of subjects in the present study. Patients with hypothyroidism and hyperthyroidism has a similar seroprevalence of *T. gondii* infection. However, stratification by age groups showed that young (50 years or less) patients had a significantly lower (1.2%) seroprevalence of *T. gondii* infection than their age- and gender-matched controls (8.6%). Furthermore, in this age group, patients with hypothyroidism has a significantly lower (0%) *T. gondii* seroprevalence than their age- and gender-matched controls (9.0%). We are not aware of any report on a negative association of *T. gondii* seropositivity and hypothyroidism in general or in a subset of patients with specific age. It is not clear why younger patients suffering from thyroid dysfunction or hypothyroidism had a very low seroprevalence of *T. gondii* infection. This negative association was unexpected since some reports suggest that *T. gondii* infection might be positively associated with thyroid disease. Infection with *T. gondii* has been associated with autoimmune thyroid diseases [17, 18], an elevation of autoantibodies to thyroid peroxidase [19], and a mild increase of thyroid hormone production in pregnancy [20]. Furthermore, an impaired thyroid function was observed in murine toxoplasmosis [21]. The negative association between thyroid dysfunction and *T. gondii* exposure found in the present study may be interpreted as follows: that this infection has not any role in thyroid dysfunction or that this infection has a protective role against thyroid dysfunction. Concerning the former, it is possible that *T. gondii* might infect the thyroid gland in a quite few individuals infected with this parasite and therefore, any inflammation or tissue damage leading to thyroid dysfunction might be seldom observed. With respect to the latter, *T. gondii* has been negatively associated with multiple sclerosis [24], atopic diseases [25], and specific IgE against aeroallergens and skin prick test reactivity in children [26]. It is unclear whether *T. gondii* has a protective role against thyroid dysfunction in general and hypothyroidism in particular as the one observed in exposure to this parasite and other pathogens against atopy and asthma [26]. Further research is needed to confirm or challenge this protective role of *T. gondii* and to demonstrate any mechanism involved in this protection.

The limitations of the present work include enrollment of a small number of patients suffering from hyperthyroidism and of patients in only one health institution. The heterogenicity of cases was also a limitation in the current study. The seroprevalence of *T. gondii* infection was compared in age groups with a wide range of years. In addition, no data on the length of evolution of the thyroid disfunctions was obtained. Further research with a larger number of patients with hyperthyroidism, homogeneity of cases, comparison of seroprevalence in age groups with small range of years, with information about the length of evolution of thyroid disfunction and performed in several health institutions is needed.

## Conclusions

Our results suggest that thyroid dysfunction is not associated with seropositivity to *T. gondii* in general; however, in young (50 years or less) patients, a negative association between infection and thyroid dysfunction and hypothyroidism was found. Further research to confirm this negative association is needed.

## Supplementary information

**Additional file 1.** Dataset of cases and controls. Data of cases and controls used and/or analyzed during the current study.

## Data Availability

The dataset used and/or analyzed during the current study is available as Additional file 1.

## Abbreviations

CI: Confidence interval
IgG: Immunoglobulin G
OR: Odds ratio
*T. gondii*: *Toxoplasma gondii*
